# Can the efficacy of subacromial corticosteroid injection be improved using a single-session mobilization treatment in subacromial impingement syndrome? A randomized single-blind controlled trial

**DOI:** 10.3906/sag-1909-51

**Published:** 2020-02-13

**Authors:** Fazıl KULAKLI, İlker İLHANLI, İlker Fatih SARI, Adem TÜRKÖZ, Canan ÇELIK

**Affiliations:** 1 Department of Physical Medicine and Rehabilitation, Faculty of Medicine, Giresun University, Giresun Turkey

**Keywords:** Subacromial impingement syndrome, mobilization, corticosteroid injection

## Abstract

**Background/aim:**

The objective in this study is to assess the short-term effects of a single-session mobilization in addition to subacromial corticosteroid (SACS) injection in impingement syndrome.

**Materials and methods:**

The study was designed as a prospective randomized controlled single-blind, parallel group clinical trial. Patients (totally 84) were divided randomly into two groups equally. Forty-two patients in Group 1 received mobilization and SACS injection, whereas 42 patients in Group 2 only received SACS injections. A single SACS injection was applied in all patients. Mobilization was administered as a single session right after SACS injection. Patients’ evaluations were performed measuring active range of motion (AROM), visual analogue scale (VAS) during activity and rest, and Disabilities of Arm, Shoulder, and Hand Score (DASH) prior to treatment and in the first and fourth weeks following the treatment.

**Results:**

Both groups showed significant improvement in terms of AROM, VAS, and DASH scores in each evaluation step (P < 0.05). Visual analogue scale activity in the first week was significantly better in Group 1 (P = 0.028). Also, flexion and abduction degrees showed significantly better outcomes in Group 1 (P = 0.007, P = 0.036).

**Conclusion:**

Addition of single-session mobilization might provide rapid improvement in flexion and abduction as well as early pain relief following SACS injections.

## 1. Introduction

Shoulder pain is a common problem in the general population. About half of the population suffers from shoulder pain and the most frequent cause of shoulder pain is defined as subacromial impingement syndrome (SIS) [1,2]. In this condition, the shoulder pain seen in arm elevation is due to the narrowing of the space between the coracoacromial arch and the humerus, trapping the subacromial/subdeltoid bursae, rotator cuff tendons, and the long head of the biceps. Main treatment goals are reducing the pain and improving shoulder function [3]. The first line of treatment includes conservative treatment modalities such as nonsteroidal antiinflammatory drugs (NSAIDs) and physical therapy [4]. In addition, alternative noninvasive treatment methods such as subacromial corticosteroid (SACS) injections, exercise, application of heat and electricity, elastic therapeutic taping, acupuncture, and manual therapy (MT) were also recommended in the previous studies on this subject [5]. 

Subacromial corticosteroid and local anesthetic injections were demonstrated both as a diagnostic and treatment method in SIS [6,7]. They were found to be more effective than placebos, exercises, and physiotherapy in this condition [7,8].

Mobilization is a well-known treatment method for shoulder pain [8]. There are no previous studies about the additional effect of mobilization on SACS injection in SIS. Mobilization is a treatment method which requires adherence, continuity, time, and physician experience on the subject. Although there are virtually no side effects attributed to this method, which is a clear advantage over other treatment options, the fact that it is a time-consuming method makes this option less favored in Turkey where there is a dense patient population. For this reason, we wanted to conduct research on the question of whether a single-session of mobilization treatment which does not require follow-up and is not time-consuming can provide additional benefit to SACS injection in SIS management. Our main objective in this study is to assess the effect of a single-session mobilization treatment in addition to SACS injection on pain severity, joint mobility, and functional status in the short-term using objective testing methods.

## 2. Materials and methods

The study was designed as a prospective randomized controlled (equal randomization 1:1) single-blind, parallel group clinical trial in the Giresun University Faculty of Medicine’s Physical Medicine and Rehabilitation Department. Ethics approval for the study was obtained from the Ethics Committee of Giresun University with decision number KAEK-46. The patients included were over 18 years of age without any significant shoulder trauma, and were diagnosed with SIS (pain in deltoid insertion area, positive Neer and Hawkins–Kennedy tests, pain on shoulder abduction), supplemented by ultrasonography (USG) findings (bunching in coracoacromial arc, subacromial bursa on lateral of impingement area, subdeltoid bursa and supraspinatus, bulging of coracoacromial ligament) in our clinic between June 2017 and September 2018. All patients had shoulder X-rays from the front and the rear to eliminate bone pathologies. Patients with main complaints of shoulder pain caused by neck problems, with acromioclavicular pathology and with other primary shoulder disorders including adhesive capsulitis or suspected full-thickness cuff-tear on USG, were excluded from the study. Adhesive capsulitis was diagnosed if there was range of motion (ROM) limitation of the shoulder to all directions both active and passive with external rotation reduced by at least 50% and elevation <100° compared with the normal side in the absence of bone pathology. Patients with a history of inflammatory arthritis, fracture, infection, malignancy, ipsilateral shoulder surgery, shoulder injection, or shoulder-focused exercise program history within 6 months were excluded from the study, as were those for whom SACS injections were contradicted and patients on a surgery waiting list [9]. G Power 3.1 software was used to calculate the required sample size. Based on a power of 80% and 5% level of significance, we calculated that the total sample size required was 72 [10]. Assuming a 10% loss to follow-up, the final sample size required was 80 patients for randomization. Following exclusion, 84 patients in total were included in the study. All participants signed an informed consent form before participating in the study.

**Figure 1:**
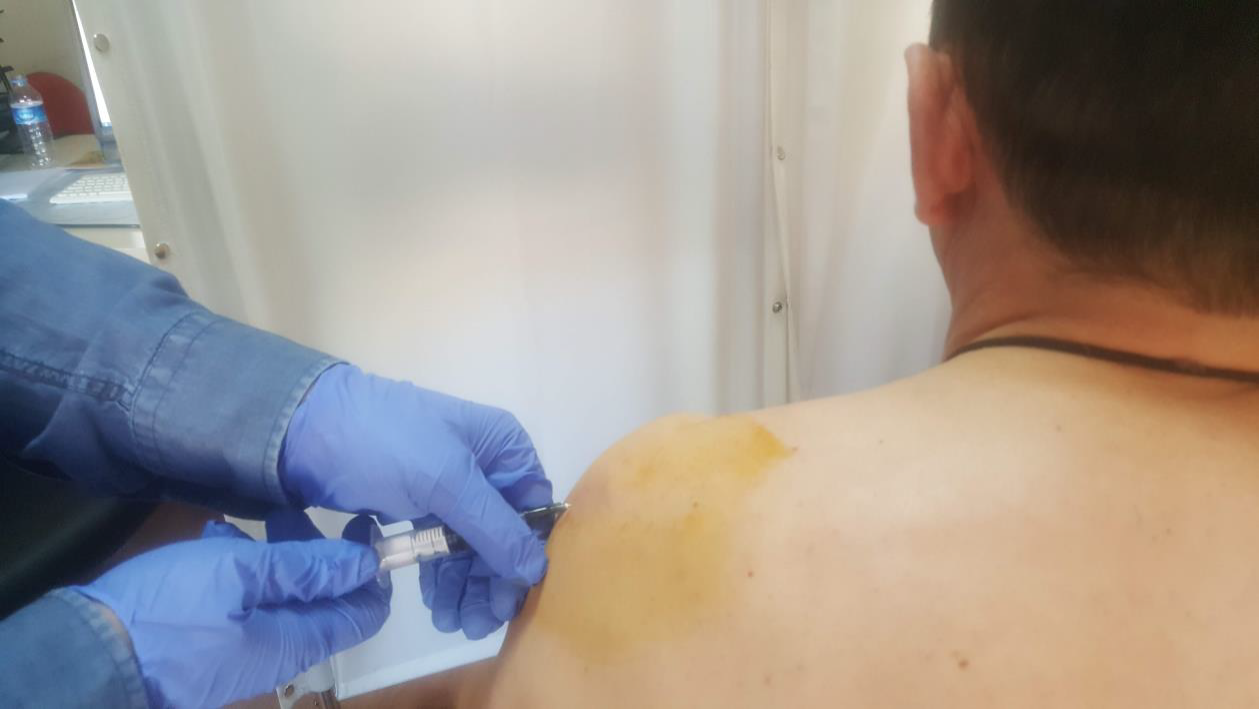
Subacromial glucocorticoid injection was applied via a palpation-guided posterolateral approach to all patients.

**Figure 2:**
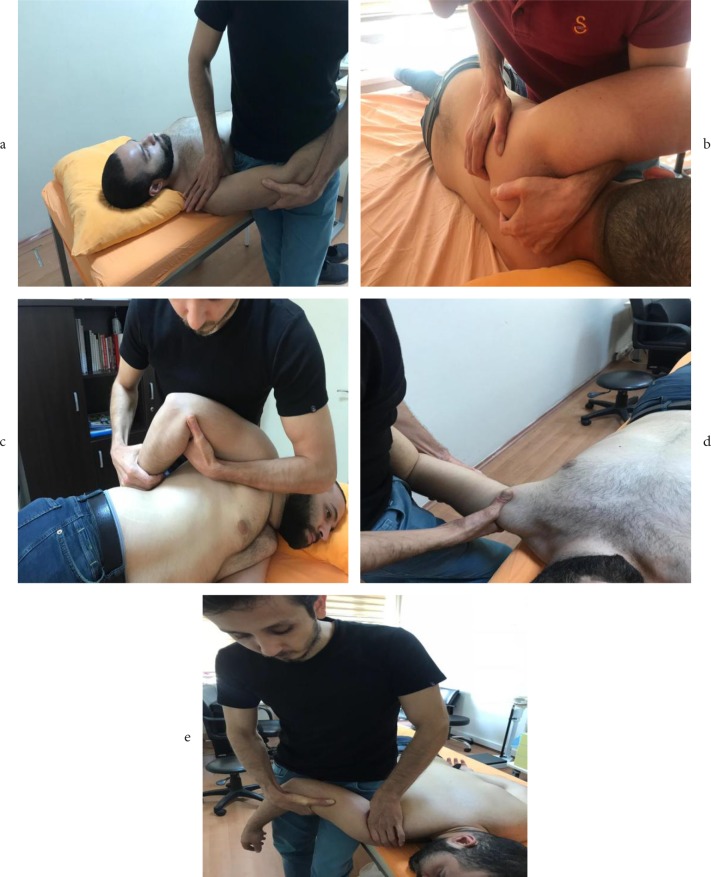
a) Anterior to posterior gliding. b) Scapulothoracic joint mobilization. c) Posterior capsule mobilization. d) Inferior gliding. e) Posterior to anterior gliding.

Patients were divided randomly into two groups equally. Group 1 consisted of 42 patients who received one session of mobilization (OSM) and SACS treatment (OSM group) whereas Group 2 (n = 42) consisted of patients who only received SACS injections (SACS group). Randomization was done using a computer with assignments placed in opaque and sequential numbered envelopes by an off-site researcher who was not involved with patient care or follow-up. A single SACS injection (20 mg triamcinolone hexacetonide) was applied via palpation-guided posterolateral approach in all patients (Figure 1). Mobilization was administered as a single session right after SACS injection. Glenohumeral anterior, posterior and inferior gliding, posterior capsule mobilization, and scapulothoracic joint mobilizations were done by a clinician who was trained and experienced in the application of this method (Figures 2a–2e). To avoid bias, clinical examination (F.K.), diagnosis (F.K.), assessment (İ.F.S.), mobilization (A.T.), and SACS injection (İ.İ.) were all performed by the same physiatrists. The researcher who reviewed pre- and posttreatment outcomes was blind to the treatment methods used on the patient.

Exercise programs (including Codman’s pendulum exercises, active shoulder ROM, and isometric strengthening exercises with limited pain) were prescribed to all patients following baseline assessment. Isotonic exercises were avoided for 2 weeks following SACS injections. Participants were instructed to rest and perform Codman’s pendulum exercises during the first 15 days. Postinjection care advice and exercise leaflets were provided to all patients. The leaflet included information about shoulder anatomy and SIS in addition to simple self-help messages about pain relief (including application of cold packs) and activities. The exercise program included a small number of standardized exercises focusing on specific muscle strengthening and ROM improvement. Patients were supervised weekly to ensure that the exercises were performed correctly and for patient adherence to the treatment. Patients were allowed to use acetaminophen and this amount was recorded. Occurrences of all potential adverse events from all interventions were also monitored closely.

The evaluation of patients was done measuring active ROM (AROM: flexion, abduction, and internal and external rotation degrees), a visual analogue scale (VAS) during activity and rest, and the Disabilities of Arm, Shoulder, and Hand Score (DASH) [11] prior to treatment and in the first and fourth weeks following the treatment. 

Pain levels during activity and rest periods were assessed using the VAS, which is a 10-cm continuous scale. The patients were asked to mark a location on the VAS scale according to their pain intensity (0 = no pain, 10 = worst pain) [12].

Active ROM degree measurement (flexion, abduction, and internal rotation and external rotation) was done with the patient seated using a plastic universal goniometer according to the evaluation method [13]. The normal shoulder flexion and abduction range was defined as 180° whereas the external an internal rotation was defined as 90° [14].

The DASH was used for functional disability assessment. The main part of DASH is a 30-item disability/symptom scale concerning the patient’s health status during the preceding week. The items evaluate the degree of difficulties seen in performing different physical tasks due to arm, shoulder, or hand issues (n = 21); the severity of each symptom such as pain, activity-related pain, tingling, weakness, and stiffness (n = 5); and the condition’s impact on social activities, sleep, and self-image (n = 4). Each item has five response options. The scores for all items are used to calculate a scale score ranging from 0 (no disability) to 100 (very severe disability) [15]. The score calculated is called the DASH score. In this study, we employed the Turkish version of DASH [16].

### 2.1. Statistical analysis

Both groups were compared in terms of demographic properties and continuous variables. In addition, differences between evaluation steps for each group were also reviewed. Baseline variable comparison between the groups was done using independent t tests or Mann–Whitney U tests for continuous variables and using the chi-square test of independence for categorical variables. Intergroup differences in mean change from baseline to each treatment period were compared using repeated measures ANOVA with adjustments for baseline levels of outcome measures. All statistical analyses were performed using SPSS 16.0 for Windows (SPSS Inc., Chicago, IL, USA). Statistical significance level was set as P < 0.05.

## 3. Results

Out of 126 patients assessed for eligibility, 84 patients met the eligibility criteria for the study and were randomized. Two patients in Group 1 and two patients in Group 2 were lost during follow-up due to personal issues. None of the participants reported an adverse effect associated with SACS injection or OSM. None of the participants came back for repeated injections during the study. Data obtained from 80 patients in total were analyzed. Demographic data analysis showed no statistically significant difference between the groups in terms of age, sex, and duration of the disease. When the occupations of the participants were examined, it was found that the blue collar workers were significantly more common in the OSM group and white collar workers were significantly more common in the SACS group (Table 1). Both groups showed significant improvement in terms of AROM, VAS pain, and DASH scores in each evaluation step (P < 0.05) (Tables 2 and 3). The VAS activity in the first week following treatment was significantly better in the OSM group (P = 0.028). Flexion and abduction degrees showed significantly better outcomes in the OSM group as well (P = 0.007, P = 0.036). Acetaminophen usage was similar in both groups (P = 0.448) and no side effects were observed in both groups during the study period.

**Table 1:** Demographic data.

		OSM group Mean ± SD	SACS group Mean ± SD	P-value
Age (years)		51.20 ±8.01	49.35 ± 9.75	0.735
Duration of complaints (months)		9.85 ± 5.54	8.65 ± 8.10	0.297
		Number (%)	Number ( %)	
Sex	Female	22 (55)	24 (60)	0.750
	Male	18 (45)	16 (40)	
Occupation	Housewife	18 (45) a	16 (40) a	0.004
	Blue collar	12 (30) a	4 (10) b	
	Retired	8 (20) a	6 (15) a	
	White collar	2 (5) a	14 (35) b	

**Table 2:** Comparison of range of motion.

		OSM group	SACS group	Group comparisons
		Degrees Mean ± SD	Degrees Mean ± SD	P-value
Flexion	Baseline	130.50 ± 37.10	132.50 ± 34.12	0.658
	1st week after treatment	146.50 ± 27.73*	140.00 ± 29.66*	0.008†
	4th week after treatment	170.50 ± 10.26*	169.50 ± 11.74*	0.791
	Within group comparisons (P)	<0.001	<0.001	
Abduction	Baseline	121.75 ± 32.11	120.50 ± 31.15	0.736
	1st week after treatment	150.50 ± 26.33*	143.75 ± 24.66*	0.036†
	4th week after treatment	175.50 ± 8.44*	169.50 ± 9.79*	0.214
	Within group comparisons (P)	<0.001	<0.001	
Internal Rotation	Baseline	54.25 ± 12.61	51.75 ± 13.58	0.457
	1st week after treatment	66.50 ± 11.22*	62.75 ± 8.74*	0.363
	4th week after treatment	71.25 ± 9.65*	68.50 ± 7.95*	0.424
	Within group comparisons (P)	<0.001	<0.001	
External rotation	Baseline	63.50 ± 14.03	60.00 ± 16.13	0.436
	1st week after treatment	74.75 ± 12.30*	71.75 ± 8.34*	0.455
	4th week after treatment	79.25 ± 10.44*	76.00 ± 9.25*	0.396
	Within group comparisons (P)	<0.001	<0.001	

**Table 3:** Comparison of groups for VAS pain and DASH scores.

		OSM groupMean ± SD	SACS groupMean ± SD	Group comparisonsP-value
VAS rest	Baseline	4.60 ± 1.40	5.05 ± 2.32	0.384
	1st week after treatment	1.35 ± 1.24*	1.95 ± 1.27*	0.331
	4th week after treatment	0.80 ± 1.53*	0.85 ± 1.26*	0.824
	Within group comparisons (P)	<0.001	<0.001	
VAS activity	Baseline	8.50 ± 1.75	8.40 ± 1.75	0.794
	1st week after treatment	2.75 ± 1.30*	4.05 ± 1.70*	0.028†
	4th week after treatment	1.80 ± 1.55*	2.45 ± 1.65*	0.086
	Within group comparisons (P)	<0.001	<0.001	
DASH	Baseline	63.95 ± 10.80	61.40 ± 9.90	0.386
	1st week after treatment	60.75 ±10.25*	58.05 ± 8.55*	0.605
	4th week after treatment	42.10 ± 12.4*	40.50 ± 10.94*	0.556
	Within group comparisons (P)	<0.001	<0.001	

* P < 0.001, 1st week after treatment vs. baseline and 4th week after treatment vs. baseline † P < 0.05, SACS+OSM vs. SACS SD: Standard deviation, VAS: Visual analogue scale, DASH: Disabilities of Arm, Shoulder, and Hand score OSM: One-session mobilization, SACS: Subacromial corticosteroid injection

## 4. Discussion

To the best of our knowledge, this is the first study that shows the additional benefits of mobilization used with SACS injection on pain, function, and ROM in SIS patients. Moreover, the significant point that differentiates our study from other studies is that the mobilization was performed as a single session right after injection. It can be argued that adding OSM to SACS injections might be beneficial in reducing pain in the acute period as compared to SACS injections alone, therefore allowing the patient a faster return to normal life. Although the OSM group showed better improvement in the short-term in terms of flexion, abduction, and pain during activity, this difference dissipated over time. Although mobilization is a safe and reliable treatment method with almost no side effects at all, it is not very popular among physicians in Turkey due to its time-consuming nature, which is not very practical in a very dense patient population. For this reason, we wanted to eliminate this time-consuming step by using OSM to supplement SACS injections to see if any other improvements can be seen with this practice. We were able to see that adding OSM to SACS injections in SIS patients caused an improvement in pain and ROM in the first week when compared to SACS injections alone. This allows the patient to return to normal life faster, eliminate workforce loss, and improve the patient’s mood due to a speedy recovery from pain and function loss due to the condition.

Subacromial corticosteroid injection is a well-known and effective method used in diagnosis and treatment for many years in SIS. Corticosteroids, such as triamcinolone, reduce pain with antiinflammatory and analgesic effects [17]. The MT and exercise are common treatment methods for SIS as a part of physical therapy. Improving function and ROM and decreasing pain are the main goals of both treatments [18,19]. A recently published metaanalysis reported that corticosteroids were superior to control and physical therapy modalities, but only during a short-term follow-up period. In addition, exercise was found to be superior to doing nothing and specific exercises were superior to nonspecific ones. The MT was superior to doing nothing for pain and MT in addition to exercise was also found to be superior to nonspecific exercise alone during a short-term follow-up period [8]. A recent trial compared SACS injection plus MT-exercise versus MT-exercise alone for SIS and determined similar improvements in pain and function at 3 months; however, in the injection group, pain and disability showed a more rapid improvement [20]. Another study divided subacromial pain syndrome patients into two groups, and treated the first group with SACS injections and the second group with SACS injections plus exercise using USG to assess patients’ conditions. The authors reported no significant additional benefit of exercise in pain severity or subacromial bursa USG appearance [21]. In our study, we found that both SACS and SACS with OSM were effective in reducing pain and increasing joint ROM in the first and fourth weeks of treatment. Although there are some studies that assess the additional benefits of exercise treatment in corticosteroid injection treatments, there are no data in other studies about the additional effect of OSM over SACS injection in SIS cases. When the additional effect of OSM was assessed in the first week of treatment, we were able to see that the ROM in flexion and abduction had an increase and VAS activity scores were better in the OSM plus SACS injection group. However, no statistically significant difference was found between the groups in terms of ROM and VAS scores in the fourth week of treatment. These results showed that rapid functional improvement might be achieved in a shorter period of time in the OSM plus SACS injection group.

Corticosteroid injections are associated with a number of side effects including infection-sepsis, skin issues (depigmentation, atrophy, hyperpigmentation), lipoatrophy, hemorrhage, postinjection flare-ups, and pain at the injection site [22,23]. In our study, none of the participants reported adverse effects associated with SACS injection or OSM.

One of the limitations of our study is that we were not able to clearly determine if the effect was due to the natural course of the illness or the placebo effect of the interventions. Another limitation is that mobilization treatment was done as a single-session treatment whereas normally it would require multiple sessions over a relatively longer period of time. As mentioned above, our main objective was to answer the question of whether we could transform mobilization treatment into an effective form that did not require longer treatment periods to be used by physicians with a limited amount of time. Finally, another limitation was that we were only able to observe the effects of the treatment in the short-term. We will see if the outcomes are sustainable in the long-term in our routine follow-up period. Even though the subject number was determined by power analysis, such studies should be performed with longer series so as to avoid interpretation limitations and allow the generalization of the results. Moreover, the follow-up periods should be extended to produce more valid and informative results, as well.

In conclusion, addition of OSM might provide rapid improvement in flexion and abduction as well as early pain relief following SACS injections in SIS. It is clear that more studies with longer follow-up periods and with repeating mobilization treatment sessions are essential to clearly grasp this subject.

## Informed consent

All participants signed an informed consent form before participating in the study.

## Ethical statement

Ethics approval for the study was obtained from the Ethics Committee of Giresun University with decision number KAEK-46.
